# Screening and risk reducing surgery for endometrial or ovarian cancers in Lynch syndrome: a systematic review

**DOI:** 10.1136/ijgc-2021-003132

**Published:** 2022-04-18

**Authors:** Natalie Lim, Martha Hickey, Graeme P Young, Finlay A Macrae, Christabel Kelly

**Affiliations:** 1 The University of Melbourne Melbourne Medical School, Melbourne, Victoria, Australia; 2 Department of Obstetrics and Gynaecology, The University of Melbourne, Melbourne, Victoria, Australia; 3 Department of Obstetrics and Gynaecology, The Royal Women's Hospital, Parkville, Victoria, Australia; 4 Cancer Research, Flinders Health and Medical Research Institute, Flinders University College of Medicine and Public Health, Bedford Park, South Australia, Australia; 5 Department of Medicine, The University of Melbourne Medicine at Royal Melbourne Hospital, Parkville, Victoria, Australia; 6 Department of Colorectal Medicine and Genetics, The Royal Melbourne Hospital, Parkville, Victoria, Australia; 7 Department of Gastroenterology, The Royal Melbourne Hospital, Parkville, Victoria, Australia

**Keywords:** Lynch Syndrome II, Endometrial Neoplasms, Endometrial Hyperplasia, Ovarian Cancer, Hysterectomy

## Abstract

**Objective:**

Lynch syndrome is a hereditary cancer syndrome caused by mismatch repair gene mutations, and female carriers are at an increased risk of endometrial and ovarian cancer. The best approach to screening is not yet clear and practice varies across countries and centers. We aimed to provide evidence to inform the best approach to screening and risk reduction.

**Methods:**

A systematic search of the literature was conducted (Medline, Embase, PubMed). Studies evaluating the following were included: women with Lynch syndrome (by mismatch repair mutation or Amsterdam II criteria), screening methods for endometrial and/or ovarian cancer, intervention included endometrial biopsy, transvaginal ultrasound, or serum cancer antigen 125 (CA-125), outcomes evaluated were number of cancers and/or endometrial hyperplasia.

**Results:**

A total of 18 studies of Lynch syndrome carriers which screened for endometrial cancer using transvaginal ultrasound and/or hysteroscopy/endometrial biopsy revealed an incidence of 3.9% at the time of screening. Most (64.1%) endometrial cancers detected were from screening, with the balance detected in symptomatic women at the first screening visits, regular review, or between screening intervals. In mismatch repair carriers, the overall sensitivity of endometrial screening was 66.7%, and the number needed to screen ranged between 4 and 38 (median 7). The sensitivity of endometrial biopsy was 57.1% and the number needed to screen was 23–380 (median 78). The sensitivity of transvaginal ultrasound was 34.4% and the number needed to screen was 35–973 (median 170). Fourteen studies which screened for ovarian cancer using transvaginal ultrasound and/or CA-125 revealed an incidence of 1.3% at the time of screening and 42.9% of ovarian cancers were detected at asymptomatic screening. The sensitivity of ovarian screening was 54.6%, and the number needed to screen was 9–191 (median 23) in mismatch repair carriers. Thirteen studies reported 5.8% incident endometrial cancers and 0.5% ovarian cancers at time of risk reducing surgery.

**Conclusions:**

There is limited evidence to support screening for endometrial and ovarian cancer in Lynch syndrome and data on mortality reduction are not available. Further prospective, randomized trials comparing targeted screening methods are needed. Risk reducing surgery remains the most reliable way to reduce endometrial and ovarian cancer risk in Lynch syndrome.

**HIGHLIGHTS**

- Endometrial and ovarian cancer rates were highest in MLH1 and MSH2 carriers, respectively
- Endometrial biopsy had a sensitivity of 57.1% and the number needed to screen was 23–380 (median 78)
- Risk reducing surgery could be offered based on genetic pathogenic variant

## Introduction

Lynch syndrome is an autosomal dominant hereditary cancer syndrome caused by mutations in mismatch repair genes (*MLH1, MSH2, MSH6, PMS2*). Epithelial cell adhesion molecule (*EPCAM*) can also predispose to *MSH2* deficient cancers. Before genetic testing, patients were diagnosed with Lynch syndrome using Amsterdam II criteria based on family history.^1^

Mismatch repair carriers are at higher risk of multiple malignancies, including colorectal, endometrial, ovarian, gastric, small bowel, pancreatic, biliary, renal, bladder, prostate, skin, and brain cancers.^2^ Women have 13–47% lifetime risk of endometrial cancer, and 3–17% risk of ovarian cancer, depending on the mismatch repair gene.^2 3^ The management of gynecological cancer risk involves screening and/or risk reducing hysterectomy and salpingo-oophorectomy. The Manchester International Consensus Guidelines^4^ and Mallorca Group^5^ recommend risk reducing surgery in all mismatch repair carriers except for *PMS2* carriers, without screening, while the US Multi-Society Task Force recommends screening for endometrial cancer by annual endometrial biopsy from 35 to 40 years.^6 7^

Only one previous systematic review has evaluated the benefits of gynecological cancer screening in Lynch syndrome. This included five studies and concluded there was insufficient evidence to support screening for either endometrial or ovarian cancer.^8^ Our review provides an updated analysis of the evidence for the effectiveness of endometrial and ovarian cancer screening and risk reducing hysterectomy and bilateral salpingo-oophorectomy in cancer prevention.

## Methods

### Endometrial and Ovarian Cancer Screening

Medline (Ovid), Embase, and PubMed databases were searched in August 2020 using relevant medical subject headings and keywords (Online Supplemental Table 1). Reference lists were searched for relevant articles. The same search was updated in November 2021. Articles meeting all of the following criteria were included: women with Lynch syndrome (by mismatch repair mutation or Amsterdam II criteria), screening methods for endometrial and/or ovarian cancer, intervention included endometrial biopsy, transvaginal ultrasound, or serum cancer antigen 125 (CA-125), and those where outcomes were number of cancers and/or endometrial hyperplasia.

10.1136/ijgc-2021-003132.supp4 Supplementary data

Articles meeting any of the following criteria were excluded: personal history of endometrial or ovarian cancer, non-Lynch syndrome hereditary cancer syndromes, other screening methods, screening was not for gynecological cancers, outcomes were cost effectiveness of screening or patient perception of screening, those not published in English, or did not contain patient data.

### Risk Reducing Surgery

Medline (Ovid), Embase, and PubMed databases were searched in August 2020 using relevant medical subject headings and keywords. Reference lists were searched for relevant articles. The same search was updated in November 2021. Similar inclusion and exclusion criteria to the above were applied detailed in Online Supplemental Table 1).

## Results

### Endometrial and Ovarian Cancer Screening

The search from August 2020 to November 2021 identified 338 studies meeting the inclusion criteria. After removing 95 duplicates and excluding 194 results through abstract screening, 43 full text articles were assessed for eligibility and six abstracts were included. Eleven full text articles were included, and four additional articles were added through reference searching. Hence 21 articles were included in the analysis (PRISMA^9^ diagram presented in Online Supplemental Figure 1).

10.1136/ijgc-2021-003132.supp1 Supplementary data

Of the 21 studies included, nine were retrospective and 12 were prospective (Online Supplemental Table 2). Eleven studies screened for both endometrial and ovarian cancers (Table 1). The age of patients screened ranged from 18 to 84 years. All studies used transvaginal ultrasound, and additional screening methods included CA-125 (12 studies), routine endometrial sampling (14 studies), and routine hysteroscopy (four studies) (Table 1).

10.1136/ijgc-2021-003132.supp5 Supplementary data

**Table 1:** Screening programs for gynecological cancer and participant characteristics across studies which studied outcomes of gynecological cancer screening in female Lynch syndrome carriers

Authors	Recommended age to commence screening (years)	Age (mean or median (range)) (years)	MMR mutation carrier status (%)	Cancers screened for	Screening method	Screening interval
Dove-Edwin et al^10^	30–35	UK: 40 (24–64) Netherlands: 42 (23–68)	AC: 171 Non-AC/AC-II: 98	EC	TVUS	1–2 years
Rijcken et al^11^	30–35	37 (27–60)	MMR: 11 (27%)	EC +OC	GE+TVUS + CA-125; curettage if positive TVUS	Annually
Renkonen-Sinisalo et al^12^	30–35		MMR: 175 (100%)	EC +OC	Varied between institutions. GE+TVUS + CA-125 +EB	2–3 years
Lecuru et al^13^	Not provided	42	MMR: 13 (21%) AC-II: 49 (79%)	EC	EB+hysteroscopy	Annually
Gerritzen et al^14^	30	46 (23–72)	MMR: 67 (67%) No mutation: 21 (21%) Unknown: 12 (12%)	EC +OC	GE+TVUS + CA-125 +ES if indicated; routine ES from 2006	Annually
Jarvinen et al^15^	35	MMR carriers: 36 (18–72) Non-carriers: 42 (18–72)	MMR: 103 (100%)	EC +OC	TVUS +EB	2–3 years
Lecuru et al^16^	30	42.5	MMR: 14 (24%) AC-II: 44 (76%)	EC	GE+TVUS + EB	Annually
Guillen-Ponce et al^17^	Not provided	Not provided	Not provided	EC	GE+TVUS; EB if TVUS abnormal	Not provided
Bats et al^18^	Not provided	41	Not provided	EC	GE+pelvic US+hysteroscopy; EB reference standard	Not provided
Arts-De Jong et al^28^	30	Not provided	MMR: 123 (87.9%)	OC	TVUS +CA-125	Annually
Manchanda et al^19^	30	43	MMR: 16 (39%) AC-II: 25 (61%)	EC	TVUS +EB+hysteroscopy	Annually
Stuckless et al^20^	Not provided	36	MSH2: 54 (100%)	EC +OC	TVUS +CA-125+EB	Not provided
Helder-Woolderink et al^21^	30	Period I: 38 (26–61) Period II: 41 (23–67)	MMR: 44 (59%) EPCAM: 3 (4%) First degree relatives: 28 (37%)	EC +OC	TVUS +CA-125. ES and hysteroscopy if indicated, routine ES from 2008	Annually
Douay-Hauser et al^22^	30	Not provided	Not provided	EC	GE+EB+TVUS±hysteroscopy	Annually
Ketabi et al^23^	25	39 (19–78)	LS (family with confirmed MMR): 236 (27%) AC: 269 (31%) AC-like: 366 (42%)	EC +OC	GE+TVUS; EB+CA-125 if TVUS abnormal	2 years
Tzortzatos et al^24^	30	50 (24–84)	MMR: 45 (100%)	EC +OC	TVUS +CA-125+EB	Annually
Gosset et al^25^	Not provided	51	MMR: 191 (100%)	EC +OC	GE+pelvic US+EB+hysteroscopy	Annually
Nebgen et al^26^	Not provided	39.2 (25.5–73.7)	MMR: 56 (70%) EPCAM: 1 (1%) AC-II: 23 (29%)	EC +OC	GE+EB for EC; TVUS +CA-125 for OC	Annually
Rosenthal et al^29^	35	Nil age range provided for MMR carriers only	MMR: 65 (100%)	OC	TVUS +CA-125	Annually
Rosenthal et al^30^	35	Nil age range provided for MMR carriers only	MMR: 120 (100%)	OC	TVUS annually+CA-125 every 4 months	Annually and every 4 months
Eikenboom et al^27^	30–35 prior to 2016 40 from 2016	46 (21.5–75) prior to 2016 53.8 (30–71.3) after 2016	MMR: 164 (100%)	EC +OC	ES +TVUS +/- CA-125	Annually

AC, Amsterdam criteria; AC-II, Amsterdam II criteria; CA-125, cancer antigen 125; EB, endometrial biopsy; EC, endometrial cancer; ES, endometrial sampling (curettage or biopsy); GE, gynecological examination; LS, Lynch syndrome; MMR, mismatch repair; OC, ovarian cancer; TVUS, transvaginal ultrasound; US, ultrasound.

### Rates of Endometrial Cancer Detected by Screening

**Figure 1:**
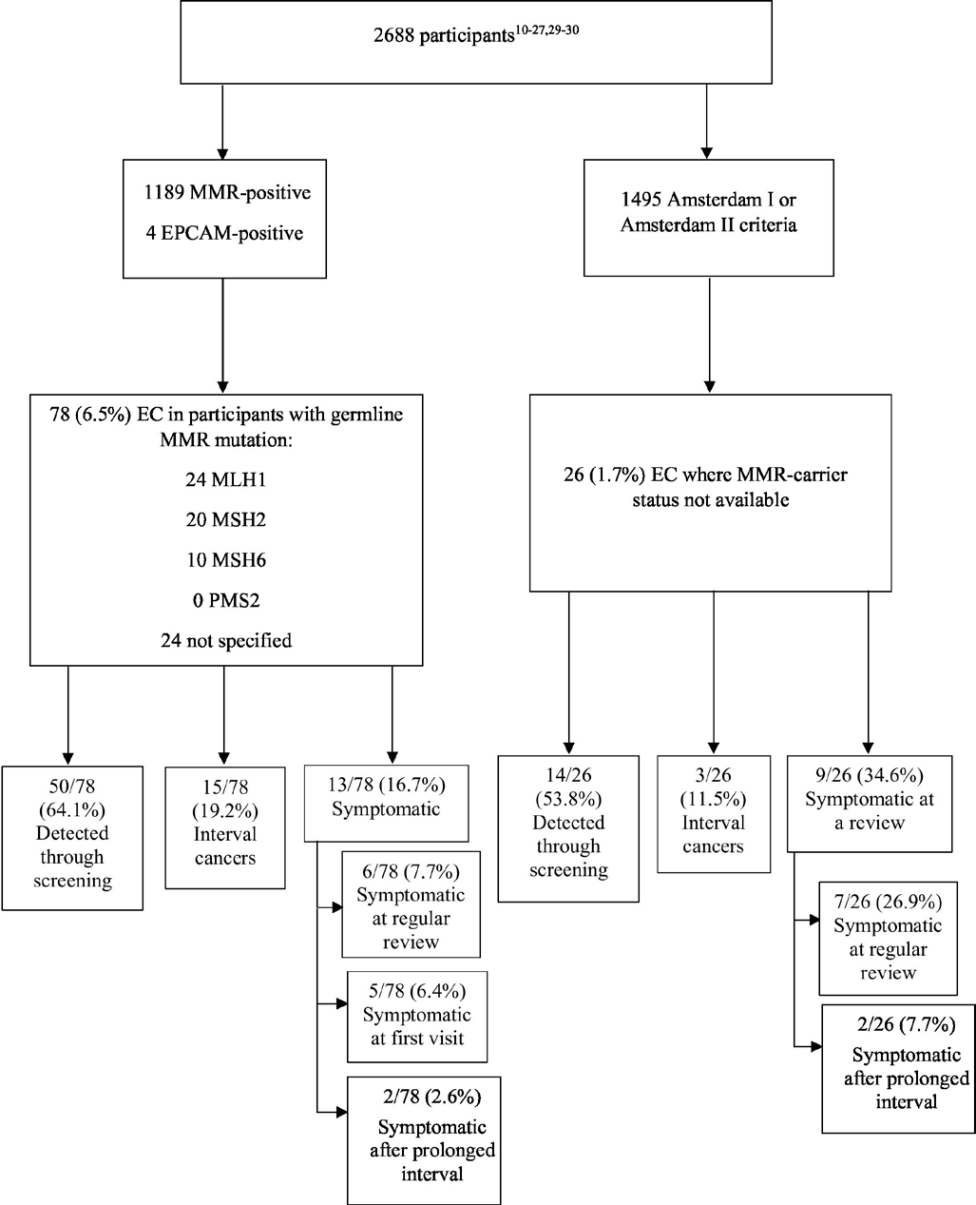
Detection of endometrial cancers (EC) through screening according to mismatch repair (MMR) carrier status. Screening methods included transvaginal ultrasound, endometrial biopsy, and hysteroscopy. There were 2688 individuals across 18 studies, 1189 of whom had a confirmed germline MMR mutation. Of MMR carriers, 6.5% were diagnosed with EC; screening detected 64.1% of these, while the remainder were diagnosed between screening intervals or presented with symptoms during a prevalent visit or regular review. 1495 participants did not have MMR carrier status available. Of these, 1.7% were diagnosed with EC; screening detected 53.8% of these, while the remainder were diagnosed at the first visit, between screening intervals, or due to symptoms.

Eighteen studies of endometrial cancer screening detected a total of 104 cancers among 2688 women (3.9%), diagnosed between at ages 36–72 years (Figure 1).^10–27^ A total of 1193 of 2688 (44.4%) patients had confirmed mismatch repair/*EPCAM* mutations and 1495 of 2688 (55.6%) were identified through Amsterdam criteria (Figure 1).

A total of 78 of 1193 (6.5%) mismatch repair/*EPCAM* carriers were diagnosed with endometrial cancer (Figure 1), representing 75% of all endometrial cancers found. Fifty of 78 (64.1%) patients were detected through asymptomatic screening at their first or subsequent screening visit, while the remainder were diagnosed due to symptoms at or between screening intervals (Figure 1). Twenty-six of 1495 women (1.7%) with Lynch syndrome diagnosed through Amsterdam criteria (or where genetic testing information was not published) were diagnosed with endometrial cancers, representing 25% of all endometrial cancers. Fourteen of 26 (53.8%) of these were through screening, while the remainder were diagnosed due to symptoms, or between screening intervals (Figure 1).

Forty of 57 (70.2%) cases of endometrial hyperplasias were found in mismatch repair carriers, 50% of which were in *MLH1* carriers (Online Supplemental Table 3). The number needed to screen, defined as the number of people needed to be screened for a diagnosis of cancer or hyperplasia, ranged between 4 and 135 (median 13) (Online Supplemental Table 4). This reduced to between 4 and 38 (median 7) when only mismatch repair carriers were included. In the mismatch repair carrier population, the sensitivity of screening to detect endometrial cancer (excluding hyperplasia) was 66.7%.

10.1136/ijgc-2021-003132.supp6 Supplementary data

10.1136/ijgc-2021-003132.supp7 Supplementary data

Combining studies from Table 2 where sufficient data were provided to inform the number of cancers or hyperplasia detected by each screening method, endometrial biopsy found 20 of 64 endometrial cancers detected via screening in total (65% stage I, 15% stage II, 5% stage III, remainder unreported) and 29 hyperplasias of 36 detected via screening in total. The sensitivity and specificity of endometrial biopsy in detecting cancer (excluding hyperplasia) were 57.1% and 97.7%, respectively. Number needed to screen, defined as the number of endometrial biopsies required to detect cancer or hyperplasia, ranged between 12 and 380 (median 19) (Online Supplemental Table 4), and between 23 and 380 (median 78) in detecting cancer only. Transvaginal ultrasound detected 11 endometrial cancers (81.1% stage I, remainder unreported) and seven cases of hyperplasia in total. Sensitivity and specificity in detecting endometrial cancer was 34.4% and 87.1%, respectively. The number needed to screen to detect either endometrial cancer or hyperplasia by transvaginal ultrasound ranged between 35 and 973 (median 89); this range remained the same to detect cancer only, however, the median increased to 170 (Online Supplemental Table 4). In studies of mismatch repair carriers only, two^12 24^ studies provided sufficient data to inform sensitivity. The sensitivity of endometrial biopsy and transvaginal ultrasound were 79.3% and 53.8%, respectively. In three studies^13 19 22^ which specified cancers detected by hysteroscopy, no additional cancers were detected when hysteroscopy was performed with endometrial biopsy.

**Table 2:** Incidence of endometrial and ovarian cancer in female Lynch syndrome carriers detected through screening, or through symptoms during interval visits or regular review

Authors	Sample size (screening visits)	No of cancers detected on final pathology (% of sample size) and age at diagnosis	No of cancers detected by screening (% of confirmed cancers)	No of interval cancers detected (% of sample size)	No of symptomatic cancers detected at screening or prevalent visit
Dove-Edwin et al^10^	269 (522)	2 (0.74%) EC Both in AC population	0 (0%) EC	2 (0.74%) EC (2 stage I)	
Rijcken et al^11^	41 (179)	1 (2.4%) EC at 61 years, MMR status not provided. 0 (0%) OC	0 (0%) EC 0 (0%) OC	1 (2%) EC (stage I)	
Renkonen-Sinisalo et al^12^	175 (503)	13 (7.4%) EC at 36-71 years, all MMR carriers (1 additional EC was not screened). 4 (2.3%) OC at 41–50 years, all MMR carriers	11 (78.6%) EC (9 stage I, 1 stage II, 1 stage III) 0 (0%) OC	2 (1.1%) EC (2 stage I) 2 (1.1%) OC (stage I, stage III). 2 (1.1%) OC incidental finding from EC surgery (both stage II)	
Lecuru et al^13^	62	3 (4.8%) EC at 37–50 years, MMR status not provided	0 (0%) EC	0 (0%) EC	3 EC presented with symptoms (3 stage I)
Gerritzen et al^14^	100 (285)	Period I: 2 (2%) EC at 52–55 years, in MMR carriers. Period II: 1 (1%) EC at 51 years, in MSH6 carrier. Unknown period: 1 (1%) OC at 50 years, MSH2	Period I: 1 (50%) EC Period II: 1 (100%) EC (both stage I) 1 (100%) OC (stage III)	0 (0%) EC	1 EC symptomatic at prevalent visit (stage III)
Jarvinen et al^15^	103 MMR carriers	19 (18%) EC at 36–72 years 6 (5.8%) OC	17 (89.5%) EC (13 stage I, 2 stage II, 2 stage III) 3 (50%) OC (2 stage I, 1 stage II)		2 EC symptomatic: 1 during screening visit; one after prolonged interval. (2 stage I) 3 OC symptomatic (2 stage I, 1 stage III)
Lecuru et al^16^	58 (96)	2 (3.4%) EC age and MMR status not provided	0 (0%) EC	0 (0%) EC	2 EC at regular review. Stages not provided
Guillen-Ponce et al^17^	91	2 (2.2%) EC	2 (0%) EC. Stages not provided		
Bats et al^18^	111	7 (6.3%) EC	7 (100%) EC. Stages not provided	0 (0%) EC	
Arts-De Jong et al^28^	140 (533)	1 (0.7%) OC at 49 years, MMR status not provided	1 (0%) OC via CA-125 at prevalent visit (stage III)		
Manchanda et al^19^	41 (69)	3 (7.3%) EC (2 MLH1 carriers, 1 unknown status). Ages 40–44 years	3 (100%) EC (3 stage I)	0 (0%) EC	
Stuckless et al^20^	54	9 (16.7%) EC at 37–54 years, in MSH2 carriers. 6 (11.1%) OC at 37-82 years, in MSH2 carriers.	5 (55.6%) EC (4 stage I, 1 stage III) 1 (16.7%) OC (stage II)	4 (7.4%) EC (3 stage I, 1 stage not provided) 2 (3.7%) OC (1 stage II, 1 not reported)	3 OC where reason for diagnosis was not reported (1 stage I, 1 stage II, 1 unreported)
Helder-Woolderink et al^21^	Total: 75 (266) Period I: 44 (117) Period II: 63 (149)	Period I: 1 (2.3%) EC at 42 years, in MSH6 (stage I) Period II: 0 (0%) EC 0 (0%) OC	0 (0%) EC	0 (0%) EC 0 (0%) OC	1 EC with symptoms (stage I)
Douay-Hauser et al^22^	157 (504)	6 (3.8%) EC	2 (50%) EC stages not provided		2 ECs at regular review. 2 ECs after 5 years disrupted follow-up
Ketabi et al^23^	871 (1945)	13 (1.5%) EC at 40–70 years, all in MMR carriers. 4 (0.46%) OC at 37-42 years, in MMR carriers	3 (23.1%) EC (2 stage I, 1 stage not provided) 1 (25%) OC (stage II)	5 (0.57%) EC (2 stage I, 2 stage II, 1 stage III) 2 (0.23%) OC (1 stage I, 1 stage III)	4 EC at regular review (all stage I); 1 EC after prolonged interval (stage IV) 1 OC (stage I)
Tzortzatos et al^24^	45	7 (15.6%) EC at 40–80 years, all MMR carriers 2 (4.4%) OC, at 38–45 years, in MSH2 carriers	3 (42.9%) EC (1 stage I, 2 stage II) 2 (100%) OC (2 stage I)	4 (8.9%) EC (3 stage I, 1 stage 1I)	
Gosset et al^25^	191 (620)	5 (2.6%) EC in MMR carriers. 1 (0.52%) OC Ages not provided	5 (100%) EC 1 (100%) OC stages not provided		
Nebgen et al^26^	80 (215)	MMR status and ages not provided	2 (7.4%) EC. Stages not provided	0 (0%) EC 0 (0%) OC	
Rosenthal et al^29^	65	3 (4.6%) OC at 35–60 years in MMR carriers	3 (100%) OC (all stage I)		
Rosenthal et al^30^	120	0 (0%) OC			
Eikenboom et al^27^	164 (680)	5 (3.1%) EC at 37–59 years in MMR carriers 1 (0.6%) OC at 48 years, in MSH2 carrier	1 (20%) EC (stage I)		4 (80%) EC (All stage I) 1 (100%) OC (stage IV)

AC, Amsterdam criteria; CA-125, cancer antigen 125; EC, endometrial cancer; EH, endometrial hyperplasia; MMR, mismatch repair; OC, ovarian cancer.

### Rates of Ovarian Cancer Detected by Screening

Fourteen studies detected 29 cancers among 2224 women (1.3%), diagnosed between 35 and 83 years old (Online Supplemental Figure 3).^11 12 14 15 20 21 23–30^ Twenty-eight of 1458 (1.9%) mismatch repair/*EPCAM* carriers were diagnosed with ovarian cancer, representing 96.6% of all ovarian cancers found. Twelve of 28 (42.9%) were in asymptomatic women detected through screening at their first or subsequent screening visit (Online Supplemental Figure 3). Of these, 11 of 12 (91.7%) were detected through transvaginal ultrasound and 3 of 12 (25%) were detected through CA-125. A total of 58.3% of ovarian cancers detected in gene carriers through screening were stage I, 25% were stage II, 8.3% were stage III, and 8.3% did not have staging reported. Eight of 28 were interval ovarian cancers which presented between regular screening visits; 50.0% were stage I, 12.5% stage II (12.5%), 25.0% stage III, and 12.5% had no reported staging. Interval ovarian cancers are defined as ovarian cancers which present clinically between regular screening visits. They are either cancers missed by screening tests at the previous screening visit, or cancers which rapidly developed between screening intervals. Two incident ovarian cancers were detected at risk reducing salpingo-oophorectomy. Eight of 28 (28.6%) patients with ovarian cancers were symptomatic: 50% were stage I, 25% stage II, 12.5% stage IV, and the remainder had no staging reported (Table 2).

10.1136/ijgc-2021-003132.supp3 Supplementary data

One (0.1%) stage III ovarian cancer was diagnosed in 766 Amsterdam criteria patients through CA-125, with no ultrasound abnormalities. The calculated sensitivity of ovarian cancer screening was 54.6% and the specificity of transvaginal ultrasound and CA-125 was 96% and 99%, respectively. The number needed to screen was 9–218 (median 72) (Online Supplemental Table 5). This reduced to 9–191 (median 23) across five studies where only mismatch repair carriers were included (Online Supplemental Table 5). There were insufficient data to calculate number needed to screen for individual ovarian cancer screening methods (transvaginal ultrasound, CA-125).

10.1136/ijgc-2021-003132.supp8 Supplementary data

### Risk Reducing Surgery

The initial search identified 438 results and the updated search in November 2021 identified 171 results, resulting in 609 total results. After removing 135 duplicates and excluding 424 reports through abstract screening, 48 full text articles were assessed for eligibility. Eleven articles were included, and two additional articles were added through reference searching. Hence 13 articles were included in the analysis (PRISMA^9^ diagram presented in Online Supplemental Figure 2).

10.1136/ijgc-2021-003132.supp2 Supplementary data

Of the 13 studies included, three were prospective cohort studies^31–33^ and 10 were retrospective histopathological analyses^24 27 34–41^ (Online Supplemental Table 6). Eight studies reported preoperative screening results. The total sample size across all included studies was 450, of whom 433 of 450 (96%) were germline mismatch repair/*EPCAM* carriers. Participants were aged 20–77 years at risk reducing salpingo-oophorectomy (Table 3). Only one study^37^ included symptomatic women (n=7) (1.5%) (Table 3).

10.1136/ijgc-2021-003132.supp9 Supplementary data

**Table 3:** Incidence of endometrial cancer or hyperplasia and ovarian cancer in female mismatch repair carriers undergoing risk reducing surgery

Author	Sample size	Participant characteristics (%)	Women with personal history of cancer (%)	Age (years) (median (range)) at RRS	RRS type	Preoperative evaluation	No of cancers (% of sample size)	No of hyperplasias (% of sample size)
Schmeler et al^31^	61	MMR: 61 (100%)	Not provided	41 (20–63)	47 (77%) TH-BSO 14 (23%) TH	Not performed	3 (4.9%) EC. Genes not provided. 38, 48, 58 years. 0 (0%) OC	
Lachiewicz et al^34^	24	MMR: 20 (83%) AC I/AC II/AC-like: 4 (17%)	Not provided	47 (32–61)	22 (92%) TH-BSO 1 (4%) BSO 1 (4%) SCH-BSO+LEEP	ES: 3/24 results available; 1 EC misdiagnosed as CAH, 2 correctly diagnosed as normal. TVUS: OC patient had ovarian cysts on preoperatively	3 (12.5%) EC. Ages not provided. MLH1, MSH2, MSH6. 1 (4.2%) OC. Age not provided, MSH2	
Karamurzin et al^35^	25	MMR: 20 (80%) AC-II: 5 (20%)	22 (88%)	48 (36–61)	18 (72%) TH-BSO 2 (8%) TH 5 (20%) TH-BSO +colectomy	ES: 9/24 results available; all negative for CAH or EC. TVUS: 1 patient with EC had abnormal findings	2 (8%) EC in 56 years MLH1; 54 years MSH2; 44 years MSH2. 1 (4%) OC in 44 years, MSH2.	3 (12%) CAH in MLH1, MSH2, AC-II 1 (4%) SH in the patient with OC
Downes et al^36^	25	MMR: 23 (92%) EPCAM: 1 (4%) Unavailable: 1 (4%)	2 (8%) had cancer at the time of RRS	47 (34–59)	23 (92%) TH-BSO 1 (4%) TH-USO 1 (4%) TH	ES: 3/25 available; 2 correctly detected CAH	2 (8%) EC in 42 years, MSH2; 59 years MSH6. 0 (0%) OC	6 (24%) CAH in 2×MLH1, 3×MSH2, 1×MSH6
Tzortzatos et al^24^	41	MMR: 41 (100%)	Not provided	53 (40–77)	32 (78%) TH-BSO 7 (17%) TH 2 (5%) BSO	EB: 1/41 available which missed EC	3 (7.3%) EC in 49 years MLH1; 46 years MLH1; 42 years MSH2. 0 (0%) OC	1 (2.4%) CAH in MLH1
Bartosch et al^37^	39 (7 symptomatic)	MMR: 39 (100%)	31 (80%)	45 (32–73)	36 (92%) TH-BSO 1 (3%) TH-USO 2 (5%) TH	EB: 10/39 available; 3 EH, 1 of which was actually EC	3 (7.7%) EC, all asymptomatic, in 50 years MLH1; 44 years MLH1; 47 years MSH2. 0 (0%) OC	6 (15.4%) EH; 4 were atypical. Atypical: 3×MLH1, 1×MSH2. Non-atypical: 2×MSH2
Wong et al^38^	27	MMR: 22 (81%) Basis of LS diagnosis unspecified: 5 (19%)	Not provided	49 (36–61)	25 (92%) TH-BSO 1 (4%) TH-USO 1 (4%) TH	ES: 12/27 available; 1 CH +11 normal IOE gross: 15/27 available; 8 abnormal including EC IOE histology: 14/27 available; 0 malignancies found (specimen with EC was not sent)	1 (3.7%) EC in 57 years, mutation positive, specific gene unavailable	2 (7.4%) CAH both in MSH2
Fedda et al^39^	29	MMR: 28 (97%) Unavailable: 1 (3%)	18 (62%)	50 (34–69)	28 (97%) TH-BSO 1 (3%) TH-BS	EB: 11/29 available; 3 correctly diagnosed EH, 1 EH misdiagnosed as EC	0 (0%) EC	5 (17.2%) EH; 4 were atypical. Atypical: 2× MLH1, 1×MSH2, 1×MSH6 Non-atypical: MLH1
Pistorius et al^40^	4	MMR: 3 (75%) AC: 1 (25%)	4 (100%)	(47–59)	4 (100%) TH-BSO	TVUS: 4/4 normal	2 (50%) EC. 49 years, AC-II; 47 years, MSH2	
Piedimonte et al^32^	41	MMR: 41 (100%)	Not provided		Not provided		0 (0%) EC	3 (20%); all atypical
Rush et al^33^	15	MMR: 15 (100%)	1 (6.7%)	47 (38–68)	15 (100%) BSO±TH	Not reported	0 (0%) EC 0 (0%) OC	0 (%)
Duenas et al^41^	66	MMR: 66 (100%)	33 (50%)	49 (36–72)	57 (86.4%) TH-BSO 8 (12.1%) TH 1 (1.5%) TH +salpingectomy	All 6 women diagnosed with cancer had normal screening prior to RRS	6 (9.1%) EC, all asymptomatic 0 (0%) OC	
Eikenboom et al^27^	53	MMR: 53 (100%)	Not provided	51	Exact number not provided for RRS patients only		1 (1.5%) EC in MSH6	

Number needed to treat is defined as the number of patients needed to undergo risk reducing surgery to detect endometrial/ovarian cancer or hyperplasia

AC, Amsterdam criteria; BS, bilateral salpingectomy; BSO, bilateral salpingo-oophorectomy; CAH, complex atypical hyperplasia; CH, complex hyperplasia without atypia; EB, endometrial biopsy; EC, endometrial cancer; EH, endometrial hyperplasia; ES, endometrial sampling; IOE, intraoperative evaluation; LEEP, loop electrosurgical excision procedure; LS, Lynch syndrome; MMR, mismatch repair; OC, ovarian cancer; RRS, risk reducing surgery; SCH, supracervical hysterectomy; SH, simple hyperplasia; TH, total hysterectomy; USO, unilateral salpingo-oophorectomy.

**Figure 2:**
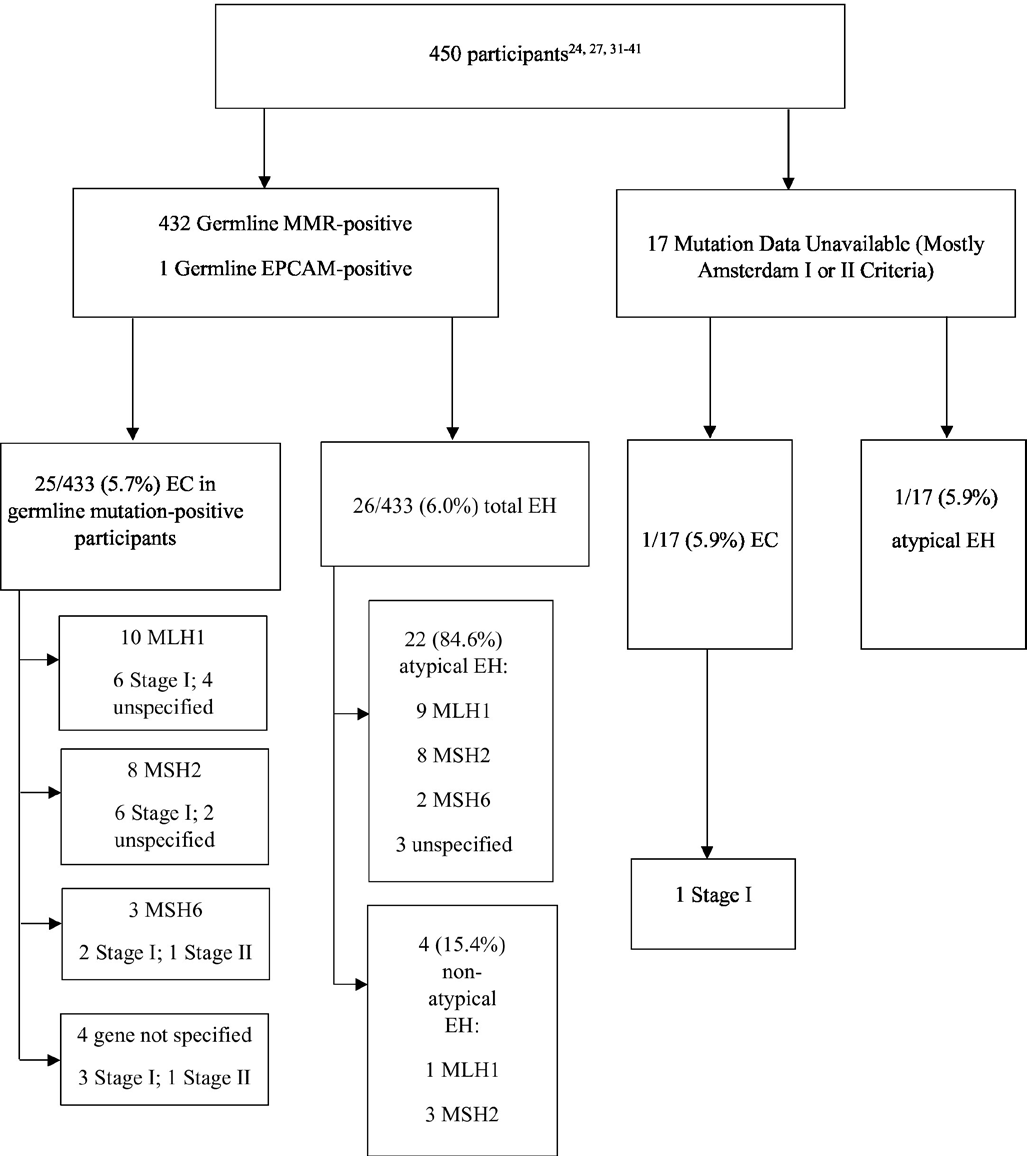
Rate of endometrial cancer (EC) or endometrial hyperplasia (EH) in prophylactic specimens from risk reducing surgery in Lynch syndrome carriers according to mismatch repair (MMR) carrier status. Of 450 participants, 433 had germline mutations, of whom 5.7% had EC at the time of risk reducing surgery; 6.0% had EH, 84.6% of which were atypical. Germline mutations and cancer stages are listed where specified.

Twenty-six of 450 women were diagnosed with endometrial cancer at the time of risk reducing hysterectomy (5.8%), aged 38–59 years (Figure 2). Twenty-seven of 450 (6.0%) patients had endometrial hyperplasia, at ages 35–53 years, 23 of 27 (85.2%) of which were atypical (Figure 2). Two of 413 (0.5%) ovarian cancers were detected at risk reducing salpingo-oophorectomy, both in *MSH2* carriers. One was 44 years old and the other patient’s age was not provided.

## Discussion

### Summary of Main Results

#### Endometrial Cancer Screening

In the 18 studies, the incidence of endometrial cancer was 3.9% over a median screening duration of 4.5 years, compared with 13–47% lifetime incidence reported in the Prospective Lynch Syndrome Database.^3^ In addition to cancer, both atypical and non-atypical hyperplasia were included as pre-malignant lesions. Atypical hyperplasia has a 27.5% cumulative risk of progression to cancer in the general population, compared with 4.6% for non-atypical hyperplasia.^42^ Despite the lower cumulative risk, we included non-atypical hyperplasia as it occurs with mismatch repair protein deficiency,^43^ and represents an opportunity for risk reduction.

More cancers were detected by screening in mismatch repair carriers compared with those who were clinically diagnosed. This may be due to a lower prevalence, more targeted screening or frequent medical reviews in carriers, or a higher index of suspicion in use of screening. Also, mismatch repair carriers were older on average than those who were not genetically tested (51 years vs 44.5 years). No endometrial cancers were diagnosed in *PMS2* carriers, consistent with findings from the Prospective Lynch Syndrome Database (12.8%), suggesting that screening is not indicated in this population.

Interval cancers, defined as cancers which present between regular screening visits, are either cancers missed by a screening method at a previous screening visit or cancers which rapidly develop between screening visits and hence were not detected previously. Interval cancers were detected in 14.3% of studies of annual screening^20 24^ with transvaginal ultrasound and biopsy, and most (80%) were stage I. No interval cancers occurred where screening included hysteroscopy.^13 18 19 21 22 25^ However, these studies had low cancer detection rates overall. The specificity of hysteroscopy alone as a screening tool cannot be determined because it was always combined with other methods.

The false negative rate for endometrial cancer screening is uncertain because there is no gold standard. However, interval cancers may indicate a false negative rate. Interval cancers could overestimate false negatives, as they will include rapidly growing de novo cancers. However, most studies reporting interval cancers used annual screening, making this less likely. Cancers presenting after missed screening visits were excluded in this sensitivity analysis. We found a 57.1% sensitivity rate for endometrial biopsy with most cancers detected early (65% stage I). However, this rate was no better than staging information available on interval cancers (72.2%), suggesting that asymptomatic screening does not detect endometrial cancers at an earlier stage than symptomatic presentations. However, not all studies reported staging. The low transvaginal ultrasound sensitivity could be influenced by a higher proportion of premenopausal women included.

Although more invasive, the specificity of endometrial biopsy is substantially higher than transvaginal ultrasound, especially in premenopausal women.^16^ False positives are more likely in premenopausal women which may lead to anxiety and over treatment. Only a small number of studies have reported sufficient data to inform sensitivity and specificity. Whether screening impacts on endometrial cancer mortality in Lynch syndrome is unknown. The 5 year survival rates from endometrial cancer in the Prospective Lynch Syndrome Database compared with sporadic endometrial cancers in the Australian population were 89% and 85%, respectively.^2 44^ Since screening is not routinely recommended in the general population, this suggests that endometrial cancer screening does not improve survival in Lynch syndrome. The number needed to screen to detect endometrial cancer varied widely. This might reflect variation in screening intervals, age at first screen (19–82 years),^23^ variable attendance at screening, and the percentage of mismatch repair carriers in each study. The number needed to screen with endometrial biopsy was less than with transvaginal ultrasounds, however, this could only be calculated for six studies, and sample sizes were small.

#### Ovarian Cancer Screening

The overall incidence of ovarian cancer was low (1.3%) and was higher among mismatch repair carriers (Online Supplemental Figure 3). As expected, all ovarian cancers detected were in *MSH2* carriers, consistent with ovarian cancer risk of 17%.^3^ Screening led to diagnosis in half of the studies (Online Supplemental Figure 3). In the only study with a control group,^20^ an equal number of participants in both the screening and control groups were diagnosed with ovarian cancer, suggesting no benefit for screening. The number needed to screen for ovarian cancer varied greatly, even in mismatch repair carriers (Online Supplemental Table 5).

The false negative rate for screening is unknown, but one-third of ovarian cancers in mismatch repair carriers were interval cancers (Online Supplemental Figure 3). In some of these cases, the cancers were diagnosed within a year after a negative screening test which would lead to false reassurance for patients, assuming they were missed at the previous screening test.^20^ Transvaginal ultrasound and CA-125 had high specificity rates based on three^11 14 21^ studies, one of which had no false positives or ovarian cancers, questioning the selection of patients for screening. False positives are common with CA-125^14,21^, which may increase the rates of unnecessary oophorectomy.^28^

#### Risk Reducing Surgery

Risk reducing surgery for Lynch syndrome includes hysterectomy with bilateral salpingo-oophorectomy. A multicenter study of 315 women (aged 20–63 years)^31^ found no endometrial or ovarian cancers after risk reducing surgery, compared with 33% endometrial and 5% ovarian cancers in controls.^31^ This study has formed the basis of guidelines recommending hysterectomy and bilateral salpingo-oophorectomy from 35 to 40 years of age or completion of childbearing.^4 6 7^

Eighteen of 26 (69.2%) endometrial cancers identified at hysterectomy and salpingo-oophorectomy were early stage (stage I), with only two stage II cancers (Figure 2). The earliest endometrial cancer was 38 years old,^31^ consistent with recommended age for risk reducing surgery.^4^ From the Prospective Lynch Syndrome Database, we would expect 59–212 endometrial cancers to be diagnosed by 70 years in 450 patients, based on a 13–47% lifetime risk, depending on pathogenic variant.^3^ A number needed to treat between 2 and 8 is calculated. This means that two surgeries are needed to diagnose one endometrial cancer in *MSH2* carriers, compared with eight surgeries in *PMS2* carriers.

In some studies, preoperative diagnoses prior to risk reducing surgery were obtained using transvaginal ultrasound or biopsy, and then compared with histopathological findings post-hysterectomy. Only 7 of 9 studies used biopsy and one study used ultrasound, limiting generalizability. Three biopsies did not correctly diagnose endometrial cancer, and one misdiagnosed hyperplasia as cancer (Table 3). These findings are consistent with those from other studies of endometrial biopsy and transvaginal ultrasound.

Ovarian cancer is much less common than endometrial cancer in Lynch syndrome (0.5% vs 5.8%), consistent with the Prospective Lynch Syndrome Database (3–17%).^3^ Based on these rates, 12–70 ovarian cancers would be expected to occur by 70 years in 413 women depending on pathogenic variant, with an estimated number needed to treat of 6–34.^3^ Six surgeries would need to occur in *MSH2* carriers to detect one ovarian cancer, compared with 34 surgeries needed in *PMS2* carriers. This aligns with recent recommendations for risk reducing salpingo-oophorectomy for *MSH2* and *MLH1* carriers and not for *MSH6* and *PMS2* carriers.^3^ In the Prospective Lynch Syndrome Database, ovarian cancer had a high 5 year and 10 year survival rate of 84%^3^ compared with 46% in the general Australian population.^44^ This could be due to the highly screened targeted population and younger age group included in the former, or the specific phenotype of Lynch syndrome cancers.

### Results in the Context of Published Literature

We found limited evidence to support ovarian cancer screening in Lynch syndrome. Similarly, the general population showed no reduction in deaths due to ovarian cancer in screened women compared with those who were not screened.^45^ Although ovarian cancer screening showed a stage shift in those at elevated risk,^30^ these were mostly in BRCA1/2 carriers who have a higher penetrance for ovarian cancer compared with mismatch repair carriers.

In Lynch syndrome, risk reducing hysterectomy and salpingo-oophorectomy is cost effective at age 40^46^ and prevents 13–45% of endometrial cancers if performed at age 40 years, and 4–18% if performed at age 60.^3^ Despite this, uptake by 50 years of age is low, at 9–28% for hysterectomy and 13–26% for salpingo-oophorectomy,^47^ and centers across the world vary in their approach to recommending risk reducing surgery.^48^ Female Lynch syndrome carriers regard cancer surveillance to be very important, and would opt for more regular investigations if offered.^49^ For those who have not completed childbearing, contact with a gynecologist as part of their multidisciplinary care could offer reassurance even without intervention. Providing information about symptoms such as abnormal bleeding and managing non-genetic risk factors for endometrial and ovarian cancer allows them to proactively manage their cancer risk. It also offers the opportunity to reinforce the use of aspirin for chemoprevention of Lynch syndrome cancers, including endometrial and ovarian cancer.^50^ Potential less invasive methods also present hope for the future in endometrial cancer screening. Cervico-vaginal cytology detected almost half of endometrial cancer cases in a meta-analysis,^51^ and vaginal and urine cytology showed high combined sensitivity.^52^ Urinary biomarkers show promise in detecting malignancy.^51 53^

### Strengths and Weaknesses

This is the first systematic review to report number needed to screen in endometrial and ovarian cancers associated with Lynch syndrome. Limitations include that approximately half the studies were retrospective, and the median sample size was only 29 women. Most had no control groups. Inclusion criteria for participants were heterogeneous with respect to family history, inclusion of mismatch repair carriers, age of onset of screening, period of follow-up, and sample size. Reduction in mortality due to screening could not be determined.

### Implications for Practice and Future Research

Adequately powered randomized controlled trials are needed to evaluate the sensitivity and specificity of each potential screening method and their effect on morbidity and mortality. The relative risks and benefits of surgery versus screening are also uncertain.

## Conclusion

Transvaginal ultrasound for the detection of endometrial or ovarian cancers does not appear to reduce morbidity in Lynch syndrome. Endometrial biopsy is more sensitive and specific with a lower number needed to screen. However, endometrial biopsy is invasive. Risk reducing hysterectomy and bilateral salpingo-oophorectomy is the mainstay of prevention in Lynch syndrome but recommendations vary depending on the mismatch repair gene.

## Data Availability

Data are available upon reasonable request. Data used to calculate sensitivity, specificity, or number needed to screen are available on request.
