# Supplementary material for: Screening and risk reducing surgery for endometrial or ovarian cancers in Lynch syndrome: a systematic review
Source: Int J Gynecol Cancer. 2022 Apr 18;32(5):646–55. doi: 10.1136/ijgc-2021-003132 (PMC9067008; doi:10.1136/ijgc-2021-003132)
Supplement: Supplementary data [file ijgc-2021-003132supp004.pdf]

Supplemental Table 1. Search strategies for screening and risk-reducing surgery

**Screening Search Strategy**	**Risk-Reducing Surgery Search Strategy**
"Lynch Syndrome" or "Hereditary Non-Polyposis Colorectal Cancer" or "Mismatch Repair"	
AND	
"Gyn*ecologic* cancer" or "Endometrial cancer" or "Ovarian cancer" or "Endometrial hyperplasia"	
AND	
"Screening" or "Surveillance" or "Endometrial biopsy" or "Transvaginal ultrasound" or "CA-125"	"Risk reducing surgery" or "Prophylactic surgery" or "Hysterectomy" or "Salpingo-oophorectomy" or "Oophorectomy"
	Limited to 2010-current
Limited to English Language	

Inclusion and exclusion criteria for studies on risk-reducing surgery:

Articles meeting all of the following criteria were included: 1) The population included women with Lynch syndrome either with a confirmed MMR mutation or who fulfilled Amsterdam II Criteria 2) The intervention was risk-reducing hysterectomy and/or salpingo-oophorectomy 3) The outcome was endometrial or ovarian cancer incidence at the time of risk-reducing surgery in prophylactic specimens 4) The results were histological findings in prophylactic hysterectomy, salpingectomy, or oophorectomy specimens.

Articles meeting any of the following criteria were excluded: 1) The population was not Lynch syndrome carriers but other hereditary cancer syndrome carriers 2) The outcomes were patient perception of the procedure 3) The outcomes were side effects about the procedure 4) The study was a review and not original data 5) The study was about cost-effectiveness of surgery 6) The article summarised guidelines in managing women predisposed to Lynch syndrome with risk-reducing surgery.
