# Supplementary material for: Screening and risk reducing surgery for endometrial or ovarian cancers in Lynch syndrome: a systematic review
Source: Int J Gynecol Cancer. 2022 Apr 18;32(5):646–55. doi: 10.1136/ijgc-2021-003132 (PMC9067008; doi:10.1136/ijgc-2021-003132)
Supplement: Supplementary data [file ijgc-2021-003132supp001.pdf]

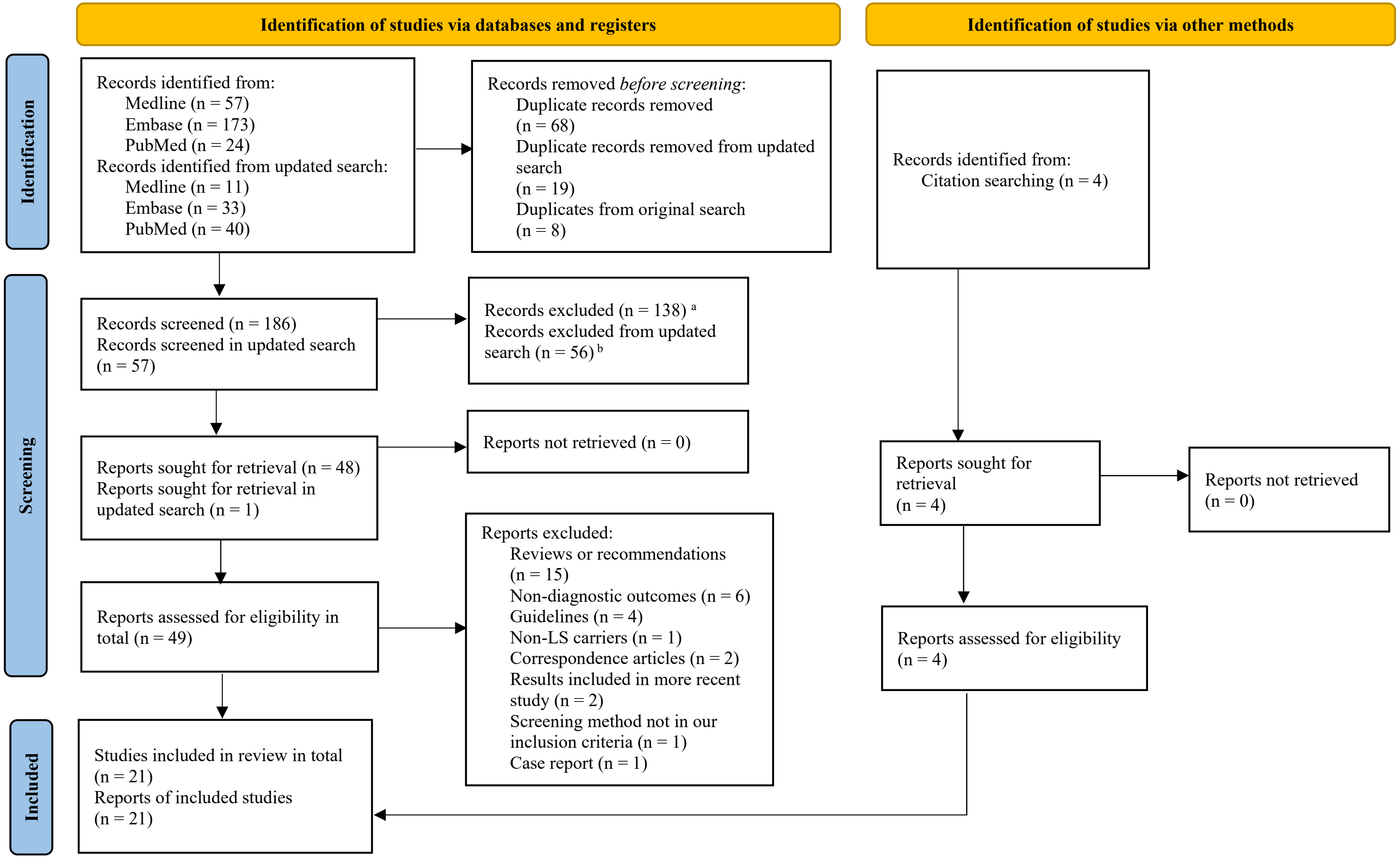

Supplemental Figure 1. PRISMA diagram for the search strategy on the effectiveness of screening for gynaecological cancer in Lynch syndrome carriers.

338 search results were identified through Medline, Embase and PubMed in total. After removing duplicates, 243 were eligible for title and abstract screening. 21 were selected for the final synthesis. Reasons for exclusion of the 194 records are stated in the text below, and reasons for exclusion of the 32 eligible articles is listed in the box.

[a] Of 138 articles excluded based on title and abstract, 22 were in a population of women already diagnosed with cancer; 22 did not include the screening methods in our inclusion criteria; 16 studied only non-gynaecological cancers; 18 studied non-diagnostic outcomes; 11 articles were about germline or tumour gene testing; 31 were reviews, recommendations, or case reports; eight studied other hereditary cancer syndromes; six were conducted in a diagnostic not screening setting; two articles reported results which were included in a more recent study; two where full data was not published and abstracts contained insufficient data.

[b] Of the 56 articles excluded based on title and abstract from the updated search, 32 studied pre-existing cancer patients; ten were reviews, recommendations, or case reports; seven studied non-gynaecological cancer; three studied non- Lynch syndrome genetic syndromes; two were surveys on clinical practice; two studied non-screening outcomes.
