# Supplementary material for: Screening and risk reducing surgery for endometrial or ovarian cancers in Lynch syndrome: a systematic review
Source: Int J Gynecol Cancer. 2022 Apr 18;32(5):646–55. doi: 10.1136/ijgc-2021-003132 (PMC9067008; doi:10.1136/ijgc-2021-003132)
Supplement: Supplementary data [file ijgc-2021-003132supp005.pdf]

Supplemental Table 2. Study Characteristics of the Studies on Endometrial and Ovarian Cancer Screening

Authors	Year	Study Setting	Study Design	Study Interval
Dove-Edwin et al[10]	2002	2 centres in the UK and the Netherlands	Retrospective Cohort	1986-1997 (UK)<br>1994-1999 (Netherlands)
Rijcken et al[11]	2003	1 centre in the Netherlands	Retrospective Cohort	1991-2001
Renkonen-Sinisalo et al[12]	2006	28 hospitals in Finland	Prospective Cohort	1996-2005
Lecuru et al[13]	2008	1 teaching hospital in France	Prospective Cohort	1999-2006
Gerritzen et al[14]	2009	1 cancer clinic in the Netherlands	Prospective Cohort	1997-2008
Jarvinen et al[15]	2009	31 centres in Finland	Prospective Cohort	≥10 years
Lecuru et al[16]	2010	1 centre in France	Prospective Cohort	1999-2007
Guillen-Ponce et al[17]	2011	Spain	Retrospective Cohort	2005-2008
Bats et al[18]	2011	NA	Prospective Cohort	NA
Arts-De Jong et al[28]	2012	The Netherlands	Prospective Cohort	
Manchanda et al[19]	2012	1 familial gynaecological cancer clinic in the UK	Prospective Cohort	2007-2010
Stuckless et al[20]	2012	1 centre in Newfoundland, Canada	Retrospective Cohort	2006-2010
Helder-Woolderink et al[21]	2013	1 centre in Netherlands	Prospective Cohort	2003-2012<br>Period I: 2003-2007<br>Period II: 2008-2012
Douay-Hauser et al[22]	2014	1 centre in France	Retrospective Cohort	1998-2014
Ketabi et al[23]	2014	Nationwide study in Denmark	Retrospective Cohort	1991-2011
Tzortzatos et al[24]	2015	Nationwide study in Sweden	Retrospective Cohort	1994-2013
Gosset et al[25]	2017	1 centre in France	Prospective Cohort	1998-2016
Nebgen et al[26]	2019	1 centre in USA	Retrospective Cohort	2002-2018
Rosenthal et al[29]	2013	42 centres in the UK	Prospective Trial	2002-2008
Rosenthal et al[30]	2017	42 centres in the UK	Prospective Trial	2007-2012
Eikenboom et al[27]	2021	1 centre in the Netherlands	Retrospective Cohort	1993-2020

NA, Not available
