# Supplementary material for: Screening and risk reducing surgery for endometrial or ovarian cancers in Lynch syndrome: a systematic review
Source: Int J Gynecol Cancer. 2022 Apr 18;32(5):646–55. doi: 10.1136/ijgc-2021-003132 (PMC9067008; doi:10.1136/ijgc-2021-003132)
Supplement: Supplementary data [file ijgc-2021-003132supp006.pdf]

Supplemental Table 3. The incidence of endometrial hyperplasia in female Lynch syndrome carriers detected through screening, or through symptoms during interval visits or regular review.

Authors	Sample Size (Screening Visits)	Number of Hyperplasias Detected on Final Histo-Pathology (% of Sample Size)	Number of Hyperplasias Detected by Screening (% of Confirmed Hyperplasia)	Number of Interval and Symptomatic Hyperplasia
Dove- Edwin et al[10]	269 (522)	Not provided		
Rijcken et al[11]	41 (179)	3 (7%) CAH	3 (7%) CAH	
Renkonen- Sinisalo et al[12]	175 (503)	9 (5.1%) EH: 4 CAH, 1 SAH, 3 CH, 1 SH	9 (100%) EH via EB<br>5 (55.6%) EH via TVUS	
Lecuru et al[13]	62	3 (4.8%) EH, all simple hyperplasia	1 (33.3%) EH as 2 were symptomatic	
Gerritzen et al[14]	100 (285)	Period I: 1 (1%) SAH at 48yo, in MLH1.<br>Period II: 3 (3%) CAH at 45-56yo, in MMR carriers.	Period I: 1 (100%) SAH<br>Period II: 3 (100%) CAH	
Jarvinen et al[15]	103 MMR carriers	7 (6.8%) EH	Unclear	
Lecuru et al[16]	58 (96)	Not provided		
Guillen-Ponce et al[17]	91			
Bats et al[18]	111	0 (0%) EH		
Manchanda et al[19]	41 (69)	3 (7.3%) EH, 1 of which was atypical	2 (67%) EH	
Stuckless et al[20]	54			
Helder- Woolderink et al[21]	Total: 75 (266)<br>Period I: 44 (117)<br>Period II: 63 (149)	Period I: 2 (4.5%) EH at 39-48yo in MMR carriers, 1 other had no final histology results.<br>Period II: 0 (0%) EH, 1 other had no final histology results.	Period I: 0<br>(0%) EH as<br>both were symptomatic. Period II:<br>0<br>(0%) EH	

Douay- Hauser et al[22]	157 (504)	0 (0%) EH		
Ketabi et al[23]	871 (1945)	5 (0.57%) EH (4 CAH + 1 CH) at 46-52yo, in MMR carriers.	0 (0%) CAH, as all had symptoms.	2 interval CAH
Tzortzatos et al[24]	45	2 (4.4%) CAH at 48-49yo, in MLH1 carriers.	2 (100%) CAH	
Gosset et al[25]	191 (620)			
Nebgen et al[26]	80 (215)	11 (13.8%) EH: 2 SH + 5 CH + 4 CAH	11 (100%) detected in screening visits	
Eikenboom et al[27]	164 (680)	8 (4.9%) EH	2 (25%) asymptomatic	3 symptomatic<br>3 unknown if symptomatic or identified through screening

CAH, complex atypical hyperplasia; CH, complex hyperplasia; SH, simple hyperplasia; SAH, simple atypical hyperplasia; EH, endometrial hyperplasia
