# Supplementary material for: Screening and risk reducing surgery for endometrial or ovarian cancers in Lynch syndrome: a systematic review
Source: Int J Gynecol Cancer. 2022 Apr 18;32(5):646–55. doi: 10.1136/ijgc-2021-003132 (PMC9067008; doi:10.1136/ijgc-2021-003132)
Supplement: Supplementary data [file ijgc-2021-003132supp007.pdf]

Supplemental Table 4. The Number Needed to Screen to detect endometrial cancer or hyperplasia in female MMR Carriers by overall screening methods, endometrial biopsy alone, or transvaginal ultrasound alone. The number needed to screen of overall screening was calculated using sample size and number of cancers and/or hyperplasia detected. The number needed to screen of individual screening methods were calculated using the number of endometrial biopsies or transvaginal ultrasounds done, and the number of cancers and/or hyperplasia detected by endometrial biopsy or transvaginal ultrasound.

	**Overall Screening by One or More Methods**	**Screening by Endometrial Biopsy**				**Screening by TVUS**			
**Authors**	**NNS to Detect One Cancer or Hyperplasia**	**No. of Endometrial Cancers or Hyperplasia [a]/Number of Screens**	**NNS to Detect One Cancer or Hyperplasia**	**No. of Endometrial Cancers/Number of Screens**	**NNS to Detect One Cancer (excluding hyperplasia)**	**No. of Endometrial Cancers or Hyperplasia[a]/Number of Screens**	**NNS to Detect One Cancer or Hyperplasia**	**No. of Endometrial Cancers/Number of Screens**	**NNS to Detect One Cancer (excluding hyperplasia)**
Dove-Edwin et al[10]	135	*	*			0/522		0/522	
Rijcken et al[11]	10	*	*			3/Not provided	NA	0/Not provided	NA
Renkonen-Sinisalo et al[12]	8	18/372	20.7	9/372	41.4	7/473	67.5	2/473	236.4
Lecuru et al[13]	10	0/116		0/116		#	#	#	#
Gerritzen et al[14]	14	4/49	12.3	1/49	49	2/221	110.5	1/221	221
Jarvinen et al[15]	4	Not provided	NA	Not provided	NA	Not provided	NA	Not provided	NA
Lecuru et al[16]	29	0/96		0/96		0/96		0/96	
Guillen-Ponce et al[17]	46	*	*	*	*	2/Not provided	NA	0/Not provided	NA
Bats et al[18]	16	Biopsy used as reference standard	NA			Not specified/161	NA	Not specified/161	NA
Manchanda et al[19]	7	5/69	13.8	3/69	23	2/69	34.5	2/69	34.5
Stuckless et al[20]	6	Not specified	NA	Not specified	NA	Not specified	NA	Not specified	NA
Helder-Woolderink et al[21]	25	0/117		0/117		0/266		0/266	

Douay-Hauser et al[22]	26	1/380	380	1/380	380	0/410		0/410	
Ketabi et al[23]	48	*	*	*	*	2/1945	972.5	2/1945	972.5
Tzortzatos et al[24]	5	5/Not provided	NA	3/Not provided	NA	0/Not provided	NA	0/Not provided	NA
Gosset et al[25]	38	Not specified	NA	Not specified	NA	Not specified	NA	0/Not provided	NA
Nebgen et al[26]	6	13/215	16.5	2/215	107.5				
Eikenboom et al[27]	33	3/244	81.3	1/244	244	0/522		0/522	

NNS, Number Needed to Screen; NA, not available as there was insufficient information to calculate; *Study did not use biopsy or was only indicated by abnormal TVUS; #Study did not use TVUS; [a] excludes interval cancers and cancers presenting symptomatically; @Study did not screen for ovarian cancer; OC, ovarian cancer
