# Supplementary material for: Screening and risk reducing surgery for endometrial or ovarian cancers in Lynch syndrome: a systematic review
Source: Int J Gynecol Cancer. 2022 Apr 18;32(5):646–55. doi: 10.1136/ijgc-2021-003132 (PMC9067008; doi:10.1136/ijgc-2021-003132)
Supplement: Supplementary data [file ijgc-2021-003132supp003.pdf]

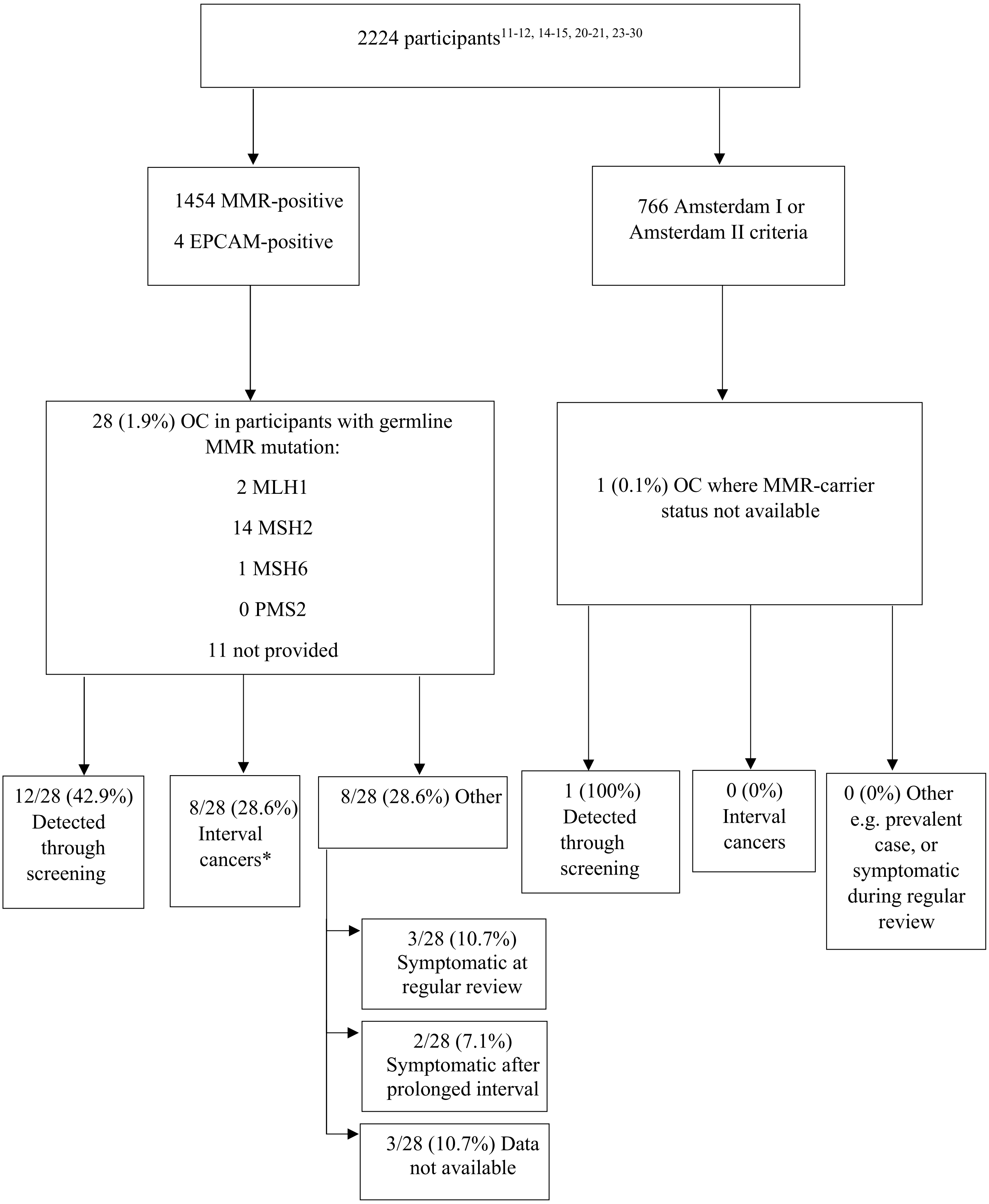
2224 participants[11-12, 14-15, 20-21, 23-30]
1454 MMR-positive
4 EPCAM-positive
766 Amsterdam I or Amsterdam II criteria
28 (1.9%) OC in participants with germline MMR mutation:
2 MLH1
14 MSH2
1 MSH6
0 PMS2
11 not provided
1 (0.1%) OC where MMR-carrier status not available
12/28 (42.9%) Detected through screening
8/28 (28.6%) Interval cancers*
8/28 (28.6%) Other
1 (100%) Detected through screening
0 (0%) Interval cancers
0 (0%) Other e.g. prevalent case, or symptomatic during regular review
3/28 (10.7%) Symptomatic at regular review
2/28 (7.1%) Symptomatic after prolonged interval
3/28 (10.7%) Data not available

Supplemental Figure 3. Detection of ovarian cancers (OC) through screening according to MMR carrier status. Screening methods included TVUS and CA-125. There were 2224 individuals across 14 studies, 1458 of which had confirmed germline MMR/EPCAM mutation. Of mutation carriers, 1.9% were diagnosed with OC, screening detected 42.9% of these, while the remainder were diagnosed between screening intervals or presented with symptoms during a prevalent visit or regular review. 766 participants did not have MMR carrier status available; only 1 case of OC (0.1%), a prevalent case, was diagnosed. *Two of these women in screening programs had incidental ovarian cancers found during surgery for endometrial cancer.
