# Supplementary material for: Screening and risk reducing surgery for endometrial or ovarian cancers in Lynch syndrome: a systematic review
Source: Int J Gynecol Cancer. 2022 Apr 18;32(5):646–55. doi: 10.1136/ijgc-2021-003132 (PMC9067008; doi:10.1136/ijgc-2021-003132)
Supplement: Supplementary data [file ijgc-2021-003132supp008.pdf]

Supplemental Table 5. The Number Needed to Screen to detect ovarian cancer in female Lynch syndrome carriers by transvaginal ultrasound and/or CA-125

Authors	NNS by Sample Size
Rijcken et al[11]	No OC detected
Renkonen-Sinisalo et al[12]	44
Gerritzen et al[14]	100
Jarvinen et al[15]	17.2
Arts de Jong et al[28]	140
Stuckless et al[20]	9
Helder-Woolderink et al[21]	No OC detected
Ketabi et al[23]	218
Tzortzatos et al[24]	23
Gosset et al[25]	191
Nebgen et al[26]	No OC detected
Rosenthal et al[29]	22
Rosenthal et al[30]	No OC detected
Eikenboom et al[27]	164
