# Supplementary material for: Screening and risk reducing surgery for endometrial or ovarian cancers in Lynch syndrome: a systematic review
Source: Int J Gynecol Cancer. 2022 Apr 18;32(5):646–55. doi: 10.1136/ijgc-2021-003132 (PMC9067008; doi:10.1136/ijgc-2021-003132)
Supplement: Supplementary data [file ijgc-2021-003132supp002.pdf]

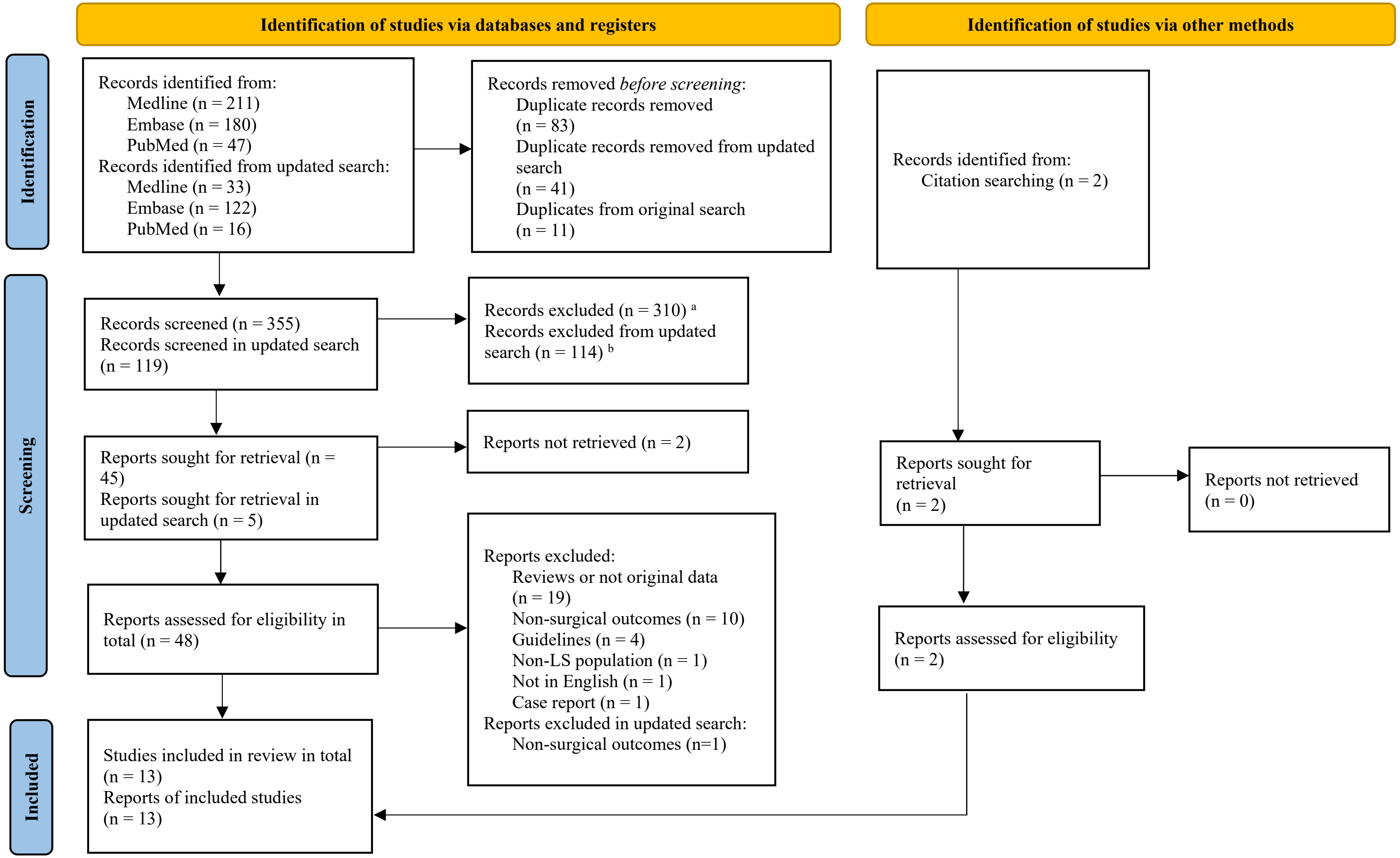

Supplemental Figure 2. PRISMA diagram for the search strategy on the rate of gynaecological cancer in prophylactic specimens from risk reducing surgery.

609 search results were identified through Medline, Embase and PubMed in total. After removing duplicates, 474 were eligible for title and abstract screening. Reasons for exclusion of the 424 records are stated in the text below, and reasons for excluding the 37 articles are listed in the figure. 13 articles were included in the final review.

[a] Of 310 articles excluded, 100 articles studied symptomatic women or those with pre-existing endometrial or ovarian malignancy; 69 articles were not about risk-reducing surgery; 53 were about cancers other than endometrial and ovarian cancer; 24 had outcomes other than surgical pathology; 22 were not restricted to MMR carriers but were studying other genetic syndromes; 15 were recommendation articles; 13 were about genetic testing or counselling; 14 were reviews, case reports or commentaries.

[b] Of 114 articles excluded based on title and abstract from the updated search, 29 studied the clinicopathology of cancer, 26 were reviews/recommendations, 31 studied women with pre-existing gynaecological cancer, 13 had outcomes other than surgical pathology, eight were case reports, four studied non- Lynch syndrome genetic syndromes, one was an article correction, one studied non-surgical prevention methods, and one studied non-gynaecological cancer.
