# Supplementary material for: Screening and risk reducing surgery for endometrial or ovarian cancers in Lynch syndrome: a systematic review
Source: Int J Gynecol Cancer. 2022 Apr 18;32(5):646–55. doi: 10.1136/ijgc-2021-003132 (PMC9067008; doi:10.1136/ijgc-2021-003132)
Supplement: Supplementary data [file ijgc-2021-003132supp009.pdf]

Supplemental Table 6. Study Characteristics of Studies on Risk-Reducing Surgery in Female Lynch syndrome Carriers

Author	Year	Study Setting	Study Interval	Study Design
Schmeler et al[31]	2006	3 centres in USA	1973-2004	Prospective
Lachiewicz et al[34]	2013	1 centre in USA	1999-2012	Retrospective
Karamurzin et al[35]	2013	1 centre in USA	2000-2011	Retrospective
Downes et al[36]	2014	1 centre in Canada	1982-2012	Retrospective
Tzortzatos et al[24]	2015	Nationwide Study in Sweden	1994-2013	Retrospective
Bartosch et al[37]	2016	3 tertiary referral centres in Portugal and USA	1995-2015	Retrospective
Wong et al[38]	2018	1 centre in USA	1990-2017	Retrospective
Fedda et al[39]	2020	1 centre in USA	2005-2018	Retrospective
Pistorius et al[40]	2006	1 centre in Germany	1995-2003	Retrospective
Piedimonte et al[32]	2021		2015-	Prospective
Rush et al[33]	2020	1 centre in USA	1999-2017	Prospective
Duenas et al[41]	2020	1 centre in Spain		Retrospective
Eikenboom et al[27]	2021	1 centre in the Netherlands	1993-2020	Retrospective
